# An Overview of Gut Microbiota and Colon Diseases with a Focus on Adenomatous Colon Polyps

**DOI:** 10.3390/ijms21197359

**Published:** 2020-10-05

**Authors:** Oana Lelia Pop, Dan Cristian Vodnar, Zorita Diaconeasa, Magdalena Istrati, Adriana Bințințan, Vasile Virgil Bințințan, Ramona Suharoschi, Rosita Gabbianelli

**Affiliations:** 1 Department of Food Science, University of Agricultural Sciences and Veterinary Medicine, 400372 Cluj-Napoca, Romania; oana.pop@usamvcluj.ro (O.L.P.); dan.vodnar@usamvcluj.ro (D.C.V.); zorita.diaconeasa@gmail.com (Z.D.); 2 Regional Institute of Gastroenterology and Hepatology “Prof. Dr. Octavian Fodor”, 400158 Cluj-Napoca, Romania; magdaistrati@yahoo.com; 3 1st Medical Clinic, Department of Gastroenterology, Emergency County Hospital, 400006 Cluj Napoca, Romania; abintintan@yahoo.com; 4 1st Surgical Clinic, Department of Surgery, University of Medicine and Pharmacy Cluj Napoca, 400006 Cluj Napoca, Romania; vasile.bintintan@umfcluj.ro; 5 Unit of Molecular Biology, School of Pharmacy, University of Camerino, Via Gentile III da Varano, 62032 Camerino, Italy

**Keywords:** microbiota, adenomatous colon polyps, colon diseases

## Abstract

It is known and accepted that the gut microbiota composition of an organism has an impact on its health. Many studies deal with this topic, the majority discussing gastrointestinal health. Adenomatous colon polyps have a high prevalence as colon cancer precursors, but in many cases, they are hard to diagnose in their early stages. Gut microbiota composition correlated with the presence of adenomatous colon polyps may be a noninvasive and efficient tool for diagnosis with a high impact on human wellbeing and favorable health care costs. This review is meant to analyze the gut microbiota correlated with the presence of adenomatous colon polyps as the first step for early diagnosis, prophylaxis, and treatment.

## 1. Introduction

Trillions of microbes inhabit the human body, and most of them are present mainly in the gastrointestinal tract. They are more numerous than all of our cells [1]. By different mechanisms that are not fully understood, the microbiota balance influences our current and future wellbeing [2]. Because of their abundance in the gut, we can affirm that all gastrointestinal diseases are in direct correlation with the gastrointestinal microbiota balance. Early identification of any unusual changes in this balance can allow incipient diagnosis, which can ensure, in most cases, successful treatment and favorable long-term prognosis. A non-invasive, cost-effective, and efficient diagnosis can be ensured, but an intense study of the microbiota pattern in different intestinal diseases must be validated.

The purpose of this review is to highlight the root of the mechanisms by which nutrients, food components, and medical interventions that spot the gut microbiota may play a role in the management of adenomatous colon polyps (ACPs). This paper will principally focus on the data relating to the effect of gut microbiota modulation and ACP prevention, amelioration, and treatment.

## 2. Human Microbiota and Colon Diseases

The diversity and abundance of specific taxa (i.e., species, genus, family) in the gut microbiota plays a key role in the modulation of human health. Typically, the human gut microbiome is dominated by five main bacterial phyla: *Firmicutes*, *Bacteroidetes*, *Proteobacteria, Actinobacteria,* and *Verrucomicrobia*. The thousands of metabolites produced by gut microbiota impact the host’s health significantly.

Alterations in the gut microbiota and its metabolites due to a diet that is poor in fiber can lead to dysfunction of the gut’s epithelial barrier, production of pro-inflammatory cytokines (i.e., Interleukin 6 (IL6), Tumour Necrosis Factor alpha (TNF-α), Interleukin beta (IL1β)), and increase of the gut’s permeability [3,4]. A high-quality diet reflects gut microbiota diversity and richness [5]. Moreover, a maternal diet and weaning influence microbiota maturation [6]; that is, the infant’s flavor perception is modulated by the mother’s diet. From prenatal life and throughout development, children can learn to enjoy the flavors of healthy foods (i.e., vegetables) [7].

A fiber-rich diet (daily range of 28–35 g for adults) maintains the integrity of the mucus layer and barrier function of the gastrointestinal tract intact. In animal models, a chronic or intermittent fiber deficiency leads to dysbiosis with erosion of the mucus layer and barrier dysfunctions that cannot be prevented by adding purified prebiotic fibers (e.g., inulin, arabinoxylan, β-glucan) [8]. Dysbiosis due to the lack of fibers in our diet can increase the mucin-degrading bacteria population (e.g., *B. fragilis*, *B. caccae,* and *A. muciniphila*). Furthermore, dysbiosis can significantly decrease both the production of short chain fatty acids (SCFAs) and their protective anti-inflammatory properties [3,9].

An unhealthy diet containing red meat, processed meat, fat, sugar, and alcohol is associated with an increased risk of colorectal cancer (CRC), which is, in most of cases, derived from ACPs [10,11,12,13,14,15]. Animal-based diets significantly contribute to changes in microbiota composition, development of inflammation, DNA damage, and impaired apoptosis when compared with plant-based diets [16,17]. Microbiota metabolites formed from the oxidation of species in high-protein diets (i.e., polyamine and ammonia), high-fat diets (hydrogen sulphide from taurine, secondary bile acids), and alcohol (i.e., acetaldehyde) contribute to the generation of reactive oxygen species and genotoxicity in the host [3]. *Bacteroides fragilis* and *Enterococcus faecalis* release enterotoxins (i.e., *fragylisin*) and reactive oxygen species contributing to DNA damage, inflammation, and injury to the epithelial barrier.

Changes in the abundance of specific bacteria have been used as a biomarker for the screening of gastrointestinal diseases including ACP, CRC, inflammatory bowel disease (IBD), and irritable bowel syndrome [18,19,20]. Gut microbiota dysbiosis has been observed in pouchitis, with an increase in *Ruminococcus gnavus, Bacteroides vulgatus,* and *Clostridium perfringens*, together with a lack of *Lachnospiraceae genera (Blautia* and *Roseburia)* [21]. Positive outcomes have been measured in adults with mild/moderate ulcerative colitis after 8 weeks of fecal microbiota transplantation (anaerobically prepared pooled stool) [22]. Enrichment of *Fusobacterium nucleatum* has been observed to induce immunosuppressive activity mediated by the inhibition of T cells in colorectal carcinogenesis [23]. A prospective cohort study on 1102 patients affected by colorectal carcinoma associates the amount of *Fusobacterium nucleatum* in colorectal cancer tissue with the tumor’s location [24]. Microbiota in colitis-associated cancer differs from that observed in subjects affected by sporadic cancer without IBD. Lower *Firmicutes* and *Bacteroidetes* with a significant increase in *Proteobacteria* was observed in colitis-associated cancer, while a reduction in *Bacteriodes* and an increase in *Fusobacteria* was identified in subjects affected by sporadic cancer [25]. Patients with Crohn’s disease and ulcerative colitis show a decreased bifidobacterial population and reduction in butyrate-producing bacteria, such as *Faecalibacterium, Eubacterium, Roseburia, Lachnospiraceae,* and *Ruminococcaceae* [26].

**Figure 1:**
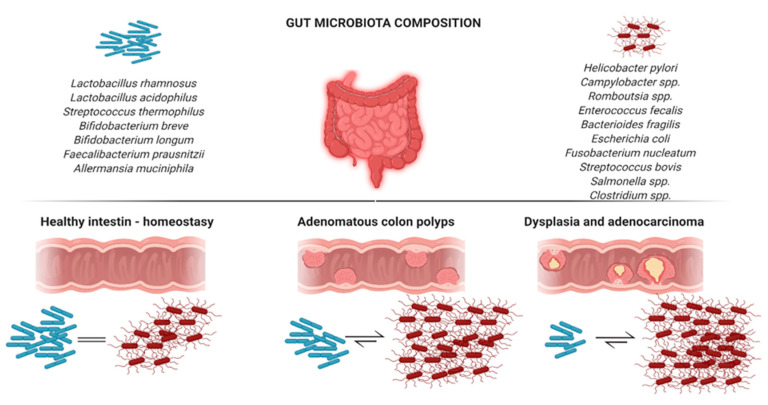
Changes in the gut microbiota composition in healthy colon, adenoma colon, and carcinoma colon.

The microbial taxa *Faecalibacterium, Bacteroides*, and *Romboutsia* were depleted in adenomatous polyps and cancerogenic mucosa [27,28]. Furthermore, a higher abundance of bacteria belonging to the *Campylobacter* genus was identified in patients affected by CRC and adenomatous polyps when compared with healthy subjects [27]. Several taxa increased (i.e., *Bilophila, Desulfovibrio*, multiple *Bacteroidetes* species), while others decreased (i.e., *Veillonella, Firmicutes*, *Clostridia*, and *Actinobacteria* family *Bifidobacteriales*) in patients with adenomas living in the United States when compared with controls. In addition, patients with dysbiosis demonstrated increased primary and secondary bile acid production and changes in sugar, protein, and lipid metabolism [29]. A graphic representation of the microbiota changes in subjects with ACP and CRC can be seen in Figure 1.

The increased interest in gut microbiota composition as the driver of gut phenotype is confirmed by numerous studies as well as by the efficacy of fecal microbiota transplantation. Although the identification of specific bacteria components is useful for diagnosis and possible therapeutic interventions, early preventive strategies aimed to counteract the development of gut dysbiosis and to maintain mucosal integrity should be considered by promoting, early in life, a daily fiber-rich and epigenetic diet containing bioactive compounds (i.e., histone deacetylase (HDAC) inhibitors) that are able to properly modulate colonocytes’ homeostasis [30].

## 3. Adenomatous Colon Polyps

In Dorland‘s Illustrated Medical Dictionary, a “morbid excrescence” is the definition given to the *polyp*. Polyps are related to adenomatous polyps because of the progress of their pathology and microscopic histology [31]. Adenomatous polyps are divided in three groups: tubular, villous, and tubule–villous (a mixture of both growth patterns). These groups depend on the grade of dysplasia, the existence of the adenocarcinoma in adenoma, and the type of histology.

**Figure 2:**
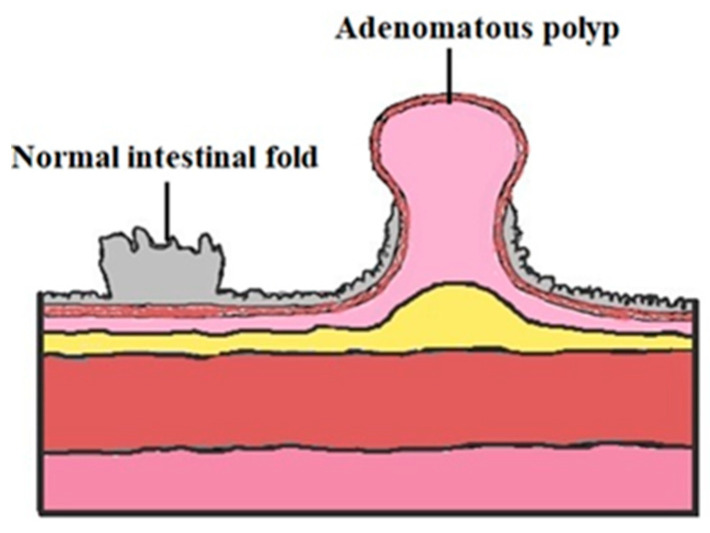
Normal intestinal fold vs. adenomatous colon polyp.

Adenocarcinomas are developed from premalignant lesions, such as ACP, which grow from a benign polyp (colonic adenoma). It is difficult to highlight the difference between an adenomatous and a normal polyp (Figure 2). However, the adenomatous type causes important modifications in the structure of the colon mucosa [32].

The average nuclear diameter of healthy mucosa is 5.6 µm. For adenoma, it is 7.44 µm [33].

During a screening colonoscopy, polyps are identified in 22.5–58.2% of patients. Some subjects with adenomatous polyps present genetic disorders of colorectal cancer [34].

Patients identified as having adenomatous polyps can be subdivided in a few categories. Familial adenomatous polyposis (FAP) (defined by numerous adenomatous colorectal polyps, from hundreds to thousands) [35] and the less harmful subcategory of attenuated FAP (AFAP) (between 1 and 100 adenomatous polyps) [36] are the first two categories. Linch syndrome produced by the mutation of MMR gene (mismatch repair) is the third group. The fourth category is familiar colorectal cancer syndrome X (FCC X), which includes people with a strong family history of bowel cancer. MUTYH-associated polyposis (MAP), caused by the MYTH gene, is another group and is spread in an autosomal recessive pattern [34].

## 4. Risk Factors for Adenomatous Colon Polyps

Several risk factors are associated with adenomatous colon polyps. The most important ones include gender, race, smoking, and obesity [37,38]. Polycyclic aromatic hydrocarbons (PAHs), which are related with tobacco carcinogens, are responsible for the appearance of an increased risk of developing ACP [39], especially when they are in a high concentration. Some studies demonstrate a link between adenomatous colon polyps and chronic obstructive pulmonary disease, even if the reason for the adenomatous polyps is smoking. According to Kearney, [37] 21 of 22 studies discovered that the risk of developing adenomatous polyps is 2–3 times higher if you smoke cigarettes for a long period of time.

Latinos and Chinese Americans have a lower risk of having these polyps than caucasians, contrasted to African Americans, who have a 1.2–2 percent higher risk. For the minority groups, the contribution in screening programs is significantly lower [40]. In 2006, about 140,000 people from America were diagnosed with bowel cancer and 56,000 died from this disease, however, most of these studies were limited to a few ethnic groups. Gender may also be a risk factor, as men have more reported cases of adenomatous polyps than women [40].

Obesity is not only correlated with metabolic and cardiovascular diseases, but also with gastrointestinal disorders as well as cancer and colon polyps [41]. According to Ahsan [42], an increased risk of ACP due to increased BMI in women (odds ratio (OR)¼ 2.1, 95% confidence interval (CI)¼ 1.1 to 4.0) was found [43].

## 5. Conventional Treatment for Adenomatous Colon Polyps

Chemoprevention is not suggested as a main treatment plan for numerous adenomatous polyps, but can be used as complementary medication. The aim is to decrease the presence of new polyps and perhaps to cause regression of the existent adenomatous polyps. The necessity of surgery can be postponed with the usage of chemoprevention and can also delay the endoscopic procedure [34].

About 80–90% of adenomatous polyps are less than 1 cm in diameter, [15] which facilitates the endoscopic removal of these polyps [44]. Polypectomy is the technique during which the polyps are eliminated in totality [45].

Examination of the colon is essential for patients who suffer from numerous adenomatous polyps if they have a contraindication for surgery or a wish to avoid this invasive procedure. Doctors should advise the patients about the progress of bowel cancer under examination. If the polyps cannot be treated by the endoscopical method, surgery should be suggested [34].

Colectomy with ileorectal anastomosis, total proctocolectomy with permanent ileostomy, or proctocolectomy with ileal pouch–anal anastomosis are surgical options for patients with serious dysplasia, adenomas larger than 5 mm, and tubulovillous adenomas [46].

## 6. Colon Cancer and Adenomatous Colon Polyps

Colon and rectal cancer are often grouped together as colorectal cancer (CRC) because of the many features that they have in common. Taken together, CRC is the third most prevalent type of cancer worldwide [47]. Its incidence appears to be higher in men than in women and much higher in developed countries. As the name suggests, CRC initially develops in the colon or rectum. Most of the time, the incipient phase of this type of cancer is represented by a growth in the deepest layer of the colon or rectum, which is the mucosal layer. These growths are called polyps. Once the polyps are formed, they can become cancerous usually in a few years. Not all polyps become cancerous. The main characteristic of a polyp that leads to its malignancy is its type. There are three main types of polyps: adenomatous polyps or adenocarcinomas, which are usually pre-cancerous and represent 96% of CRCs, as well as hyperplastic polyps and inflammatory polyps [20]. The latter are more common, but are generally not pre-cancerous. Other factors related to cancer development are the size or the number of polyps detected as well the presence of dysplasia in the polyp after surgical removal. Recently, several studies have suggested a connection between the imbalance of intestinal flora and the emergence of adenomatous colon polyps and CRC [20,48,49,50,51]. Microorganisms located in the intestines play a crucial role in food digestion, vitamin biosynthesis, and protection against pathogens. Intestinal bacterial imbalance (dysbiosis) was strongly associated with an increased risk of CRC. For instance, *Fusobacterium nucleatum* was found in high proportions in patients diagnosed with CRC [52]. These are responsible for activating a signaling pathway, especially by lowering immunity, which leads to the growth and development of tumor cells. *Escherichia coli*, another commensal microbiome of the human gut, was found to play a key role in triggering CRC [53,54]. It can induce inflammation and appears to release certain chemicals, such as cytolethal-releasing toxin (CDT) and cytotoxic necrosis factor (CNF), which can induce carcinogenesis. Enterotoxigenic *Bacteroides fragilis* [48,55] was also associated with an increased risk of CRC. It increases the level of T helper 17 (Th17) and T (Treg) cells, which promote tumor growth and development.

With these being said, CRC is commonly encountered as an aggressive form of cancer. Research suggests that human microflora plays a key role in preventing or developing colorectal cancer. Sedentarism, a diet poor in fiber, smoking, and alcohol are the extrinsic factors that can lead to colorectal cancer. A healthy lifestyle can maintain the balance of our microbiome, and thus the prevention of colorectal cancer.

## 7. Pro and Prebiotic in Human Health

The use of probiotics and prebiotics for the benefits of clinical health is a fascinating area of research that is still relevant.

Some of the best properties of probiotics, such as antipathogenicity [56,57], anti-diabetic [58,59], anti-obesity [60,61], anti-inflammatory [60], anti-cancer [62], anti-allergic [63], and angiogenic activities, as well as their effect on fatigue, the brain, and the central nervous system, have been described [64].

Moreover, prebiotics can be helpful in reducing dermatitis [65], reducing low density lipoprotein (LDL) in the blood [66], stimulating the immunological system [67], increasing iron absorption [68], maintaining the correct value of intestinal pH [69,70], and alleviating the symptoms of peptic ulcers and vaginal mycosis [2,71]. Other effects of prebiotics such as inulin and oligofructose on human health have been described as being the prevention of carcinogenesis [72,73], the support of lactose intolerance [74], and the treatment of tooth decay [75].

### 7.1. Pro- and Prebiotics in Human Microbiota Modulation

In the maintenance of human health, the gut microbiota is visualized as a symbiotic partner having a very important role. The equilibrium of the gut microbiota is correlated with factors depending on the characteristics of the host such as age, gender, and genetic circumstances. Environmental conditions such as stress, drugs, gastrointestinal interventions, and infectious and toxic agents are also important. Furthermore, the microbiota is dependent on daily dietary changes and the resistance of probiotics to environmental factors [76,77,78]. Gut microbiota composition and function is significantly influenced by the diet [79,80]. An association between the ingestion of non-digestible fibers (e.g., prebiotics) and a high number of beneficial bacteria in the gut such as *Ruminococcaceae*, *Bifidobacterium*, *Lactobacillus*, *Faecalibacterium*, and *Roseburia* is well known [79,81,82]. From an evolutionary point of view, the human species has gone through a rapid shift in habits and lifestyle. Factors such as excessive sanitation, industrialized and rich diet, sedentary behavior, and antibiotics cause an unbalance in the gut microbiota [83,84]. Many situations can lead to changes in the gut microbiota composition, which causes a need to rebalance the gut microbiota. Probiotic ingestion and probiotic foods or supplements are the most known and utilized methods, next to fecal microbial transplantation.

A study reports a fecal microbial transplantation between mother and daughter in order to treat *Clostridium difficile.* This revealed an intriguing side effect; because the donor was obese and the receiver was lean, besides the resolution of the infection, the receiver gained 16 kg (34 lbs) over the course of 16 months [85]. This is the first report of obesity as a human-to-human transmissible trait, although additional studies are required to establish the causal relevance of this report at the population level.

The human microbiota has a wide and significant influence in the metabolic processes and functions of the human body. It can have both a beneficial or detrimental impact on health, depending on its composition.

The presence of probiotics and prebiotics in the human diet can directly affect the body’s capacity to prevent, ameliorate, and reduce the prevalence of ACP. Gut microbiota metabolites play a critical function in the degeneration of adenomas to CRC, although little data about the function of most of the gut bacteria and their metabolites are available. Some of the gut’s bacteria are able to produce SCFAs such as butyrate, which can serve as energetic sources for colonic epithelial cells. A study correlated a butyrate-producing bacteria in the feces of patients with ACP, suggesting that microbial metabolites may contribute to ACP conversion to CRC [86]. A few members of the *Clostridium* genus (butyrate-producing bacteria) are capable of metabolizing primary bile acids into secondary bile acids [87,88]. These bile acids proved to have a contribution in ACP conversion to CRC by affecting the host’s metabolism and immunity [89,90,91].

Insufficient human studies have evaluated the metabolome and microbiota in relation to adenomas. Findings from a recent study suggest that there is a correlation between bacterial dysbiosis, the metabolome, and colorectal adenomas [92]. More studies are required to fully explore the correlation between microbiota, metabolome, and ACP.

The exact mechanisms of action of probiotics in the human body are currently known only partially. Probiotics have been suggested to act by inhibiting the excessive aggregation of pathogenic bacteria and preventing pathogenic host invasion, improving intestinal barrier function and interactions with receptors, and producing secretory substances such as SCFA and neurotransmitters [93].

### 7.2. Probiotic and Prebiotics in Intestinal Diseases

Dysbiosis is the state of change in microbial flora in the gut, directly causing several particular inflammatory diseases. Bowel diseases can be caused by several factors, including genetic factors, ecological factors, oxidative stress, antibiotic consumption, and weakened immune system [94,95].

Table 1 shows the cases in which probiotic interventions are used. We can observe that choosing the optimum probiotic strain can be a real challenge.

Table 2 shows the cases in which prebiotic interventions are used. We can observe that choosing the optimum prebiotic can develop the growth of probiotic strains and induce benefits.

## 8. Gut Microbiota and Their Metabolites in Adenomatous Colon Polyps

The fourth most common cancer among the population is colorectal cancer (CRC), also considered as raking third in terms of mortality [125,126]. Early screening and prevention must be implemented among those at risk owing to the high incidence of CRC and advanced adenomas.

One of the fundamental hypotheses that is widely accepted is that the adenoma-carcinoma sequence represents the steps towards the development of CRC [127,128].

Adenomatous polyps are the most common premalignant lesions in CRC [129]. It is widely believed that 40% of people over the age of 60 will develop adenomatous polyps. Furthermore, the adenomatous polyps have a 0.25% chance of transforming into cancer per year. This transformation is mainly caused by the accumulation of mutations, both somatic and germ-like. The onset trigger of this sequence of “adenoma-carcinoma” is believed to be the inactivation of the adenomatous polyposis (APC) gene [130].

The APC gene is found at 5q21-q22, which is a region containing 15 exons that code a protein with a molecular weight of 310 kDA [131]. The most frequent genetic variation in colorectal cancer was determined to be a mutation in the APC gene, with more than 3000 pathological mutations discovered so far. Most of the mutations are identified within a cluster region (MCR (mobilized colistin resistance), codons 1286–1513), often giving rise to a truncated APC protein. The implication of genetic and environmental factors as well as their interactions in the tumorigenesis of CRC have been increasingly recognized throughout the years [130,131,132].

Among environmental factors, gut microbiota and their metabolites are also present in the pathology of CRC. Nowadays, it is well known that multiple factors can alter the normal composition of the gut microbiota. It has been demonstrated that microbiotic alterations are involved in adenomas as well as in CRC owing to the metabolites resulting from fermentation of various dietary sources, which are toxic and genotoxic [130,133,134].

The microbiome of gut microbiota is different in all individuals. Gender, genetic predisposition, diet, physical activity, disease, drugs, and environmental toxicants all contribute to program human gut microbiota composition and its consequent impact on health [135,136,137]. Gut microbiota maturation starts from early life and evolves rapidly during the first 3 years of life [30,138]. Maternal transmission can influence microbiota richness and diversity. Infants born by birth canal compared with those born by caesarean section delivery show a greater microbial diversity [139]. Nursing as well as skin and oral contact with the mother influence maternal transmission. However, prenatal and postnatal antibiotic treatment can reduce microbiota diversity. Furthermore, intrapartum antibiotic exposure leads to different gut colonization when compared with postnatal treatment, and the observed impact remains stable up to 6 months of age even when lactobacilli supplementation was implemented [140]. Breastfeeding modulates gut microbiota composition in the first 4 years of life and has long-term effects on health. Longitudinal studies demonstrated that infants who received breastfeeding for 3 months have lower levels of inflammatory biomarkers (i.e., APC, IL-6) in adult age (28–32 years old) [141,142].

The human gut microorganism populations are found to be extremely complex, together making up trillions of bacteria. Change in the gut microbiota has been associated with numerous diseases, including metabolic, gastrointestinal, and neuropsychological disorders. It has been studied that the change in some gut bacteria can be determined by mutations of the host’s genes, with these changes further promoting the development of pathologies. Bacterial changes are involved in the onset apparition of precancerous lesions of the adenomatous polyp type, as well as in the accumulation of a sequence of genes during the so-called “adenoma-carcinoma sequence”. The bacterial drivers may be slowly replaced by passenger bacteria, which have a competitive advantage in the tumor niche during the development of the tumor. Therefore, early prevention of CRC as well as insight into the tumorigenesis can be observed by identifying the gut microbiota associated with the gene mutation in CRC initiation [130,133,134,143].

The relationship between the change of gut microbiota and the inactivation of APC mutations has been studied, showing that their association could potentially explain the role of gut microbiome in the transformation of APC mutant adenomatous polyps to CRC. It has been demonstrated through these studies that lower levels of *Faecalibacterium prausnitzii*, *Bifidobacterium pseudocatenulatum*, and *Ruminococcus sp 5* were found in patients with APC mutations. Higher levels of *Fusobacterium mortiferum* were also illustrated, with this deviation also being correlated with a higher occurrence of colorectal cancer [130].

There is also cumulative evidence that suggests a link between gut, colorectal adenoma, CRC, and some specific species such as *Fusobacterium nucleatum* [144,145].

Different studies have documented that an alteration of SCFAs in gut composition has also been associated with colorectal cancer, among other pathologies [146,147]. Changes in gut microbiota frequently cause a reduction in the concentration of SCFAs. These fatty acids are saturated molecules composed of one to six carbon atoms, of which acetic, propionic, and butyric acids are in the largest quantity. However, iso-butyric, valeric, and iso-valeric acids are also present in lower amounts. These fatty acids can provide energy through oxidative metabolism after entering the colonic epithelium, or they can aid in the regulation of the metabolism of fatty acids, glucose, and cholesterol upon entering the bloodstream [146,147,148].

The microbial ecosystem in the gastrointestinal tract impacts the energy metabolism mechanism because the microbiota can modulate the absorption and oxidation of macro- and micro-nutrients. Moreover, their metabolites modulate immune and metabolic responses. In the large intestine, bacteria digest dietary fiber, thus producing short chain fatty acids (SCFAs) (i.e., formic acid, acetic acid, propionic acid, butyric acid, and valeric acid) that are used by colonocytes or transported to various organs by blood circulation [149,150]. SCFAs in blood have a lower micromolar concentration range than in the colon, where it reaches 50–100 mM [151]. Butyrate, propionate, and acetate represent 15%, 25%, and 60%, respectively, of SCFAs made in the human gut [152,153].

Butyrate and propionate work as histone deacetylase inhibitors and exert a positive modulation through the activation of G-protein coupled receptors (GPRs) in colonocytes and immune cells.

Butyrate binds to GPR109A at the lumen-facing apical membrane of intestinal colonocytes and promotes anti-inflammatory responses. It can interact in a similar fashion with GPR43 and GPR41 of immune cells and with GPR41 of adipocytes [154]. Butyrate controls inflammatory responses by downregulation of the transcription factor NF-κB (nuclear factor kappa-light-chain-enhancer of activated B cells) and inhibition of pro-inflammatory cytokine release (i.e., IL6 and IL12). It also liberates anti-inflammatory cytokines (i.e., IL10). Colonocytes can use butyrate and acetate to produce β-hydroxybutyrate, a ketone body able to activate GPR109A 3–4 times more than butyrate. β-hydroxybutyrate can be carried by cells to the portal blood circulation [155]. Butyrate contributes to maintaining gut permeability; sodium butyrate supplementation upregulates genes encoding intestinal tight junction proteins in mice [156]. An important attribute of butyrate is its antineoplastic effects on human colon carcinoma cells. Cell differentiation in human colorectal cancer cell lines is induced by butyrate. It reduces the growth rate of these cell lines in vitro. Compared with healthy individuals, ACP subjects register a reduced butyrate production [157]. A possible explanation can be attributed to the fact that subjects with ACP absorb less starch than normal subjects. Starch fermentation produces higher proportions of butyrate than the fermentation of glucose or pectin [158]. McMillan’s study suggests that butyrate exhibits protection against ACP not only through its ability of inducing apoptosis in malign colon cells, but also its capacity of inhibiting the effects of secondary bile acids. Bile acids are not mutagens, but they promote tumor formation by activating protein kinase C [159]. These results are to be taken into consideration in dietary intervention protocols to reduce risk of ACP and CRC.

Acetate can also interact with GPR43 at the apical membrane of gut epithelial cells and increase the Ca^2+^ level. By K^+^-dependent membrane hyperpolarization, it is able to activate NLRP3 inflammasome (NOD-like receptor family, pyrin domain-containing, subtypes 3). The NLRP3 triggers activation of caspase-1, which promotes IL18 release, thus maintaining gut epithelial integrity in the absence of inflammation [160,161,162,163]. In his study, Weaver found significantly higher proportions of acetate and lower proportions of butyrate in the microbiota of the ACP group [164]. These findings can be related to different colonic microbial communities and fermentations that differ from those of healthy individuals. Thus, interesting findings suggest an overall higher production of SCFA’s in patients with ACP or CRC than in control groups [157,164].

Moreover, SCFAs are also important in the formation of antimicrobial peptides and in the modulation of the functions and number of regulatory T cells (Tregs), thus contributing to the inflection of host immune responses. The reduction of SCFAs’ concentration is related to unhealthy gut microbiota, thus being a cause for intestinal diseases because SCFAs are involved in their prevention by preserving the epithelial barrier functionality and by involvement in inflammatory reactions (as they regulate the transcription of proteins such as claudin-1, a tight junction molecule). SCFAs also regulate the proliferation and differentiation of colonocytes. As they increase the expression of mucin 2 and modulate immune and oxidative stress responses, these SCFAs also protect the epithelium of the large intestine [148,165,166]. The difference in proportions of SCFA between ACP or CRC patients, when compared with healthy ones, suggests differences in fermentation patterns of the colonic microbiota [164].

The researcher’s focus should not be limited only to the human gut microbiota composition in the presence of ACP, but further investigations should be done with respect to the gut microbiota metabolites. In fact, the microbiota influences the host through their metabolites. Kim and his collaborators found that, in patients with ACP, a high concentration of bioactive lipids, including polyunsaturated fatty acids, sphingolipids, and secondary bile acids, existed [92]. All these compounds are produced by the bacteria species that are dominant (in number) in patients with adenomas and CRC. This suggests that gut bacteria might contribute in the early stages of colorectal carcinogenesis and may lead to development of CRC prevention therapies, targeting early treatment.

It is thus highly suggested that the bacteria in the gut contributes in the early stages of colorectal carcinogenesis and may lead to development of CRC prevention therapies, targeting early treatment.

## 9. Summary and Conclusions

Adenomas are precursors of CRC. The imprint of the gut microbiota composition and their metabolites can provide important information about the stage of the ACP. Implicitly, new, innovative, and non-invasive treatment and amelioration protocols derive from these approaches. Currently, regarding the research on adenomatous polyps, there have been insufficient studies with respect to the gut microbiota and metabolites in patients with this pathology. Therefore, it is still not very clear how and which microbes and metabolites trigger or support the formation of ACP and further sustain the development of carcinomas.

## Figures and Tables

**Table 1:** Probiotics effect in intestinal disorders.

Intestinal Disorder	Probiotic Strain	Administration Method and Duration	Results	Reference
Constipation	*B. longum*. *B. infantis* și *B. Scurt, L. acidophilus*. *L. casei*. *L. bulgaricus*, și *L. plantarum* și *Streptococcus thermophilus*	Sachets; x 2/day for 2 weeks	Improve clinical symptoms constipation	[96]
	*L. acidophilus* (La-5) and *B. lactic* Bb-12	Yogurt; 300 g/day for 4 weeks	Improved the symptoms of constipation during pregnancy	[97]
	*B. lactis* NCC2818	Sachets; 1/day for 4 weeks	Not effective in the management of mild chronic constipation	[98]
	*S. thermophilus* MG510 and *L. plantarum* LRCC5193	Chocolate; 26 g/day for 8 weeks	Significantly ameliorated stool consistency in patients with chronic constipation	[99]
Irritable bowel syndrome (IBS)	*B. longum, B. bifidum, B. lactis, L. acidophilus, L. rhamnosus,* and *S. thermophilus*	Capsule (500 mg) of LacClean Gold-S (a multi-species probiotics); x 2/day for 4 weeks	IBS symptoms were substantially relieved (↑ 68%)	[100]
	Bio-Kult^®^ (14 different bacterial strains)	Capsule; x 2/day for 4 weeks	The change in severity and frequency of abdominal pain on the IBS-severity scoring system (IBS-SSS) The change in other gastrointestinal symptom severity scores on the IBS-SSS, Quality of Life (QoL)	[101]
	*L. paracasei*, *L. salivarius* și *L. plantarum*	Capsule; 1/day for 4 weeks	Effective in the global relief of IBS symptoms, and in relieving abdominal pain	[102]
	*L. acidophilus* DDS-1 sau *B. lactis* UABla-12	Capsule; 1/day for >6 weeks	Abdominal pain significantly improved	[103]
Ulcerative colitis (UC)	*L. salivarius, L. acidophilus*, and *B. bifidus* BGN4	1200 mg probiotic blend; x 2/day for 2 years	Combined therapy showed better improvement vs. controls. Beneficial effects of probiotics were evident even after two years post-treatment	[104]
	*L. plantarum* PL 02, *L. rhamnosus* KL 53A, and *B. longum* PL 03	Sachets; 3.0 g/day for patients with acute phase UC and 2 g/day for patients in remission for 8 weeks	Effective in inducing and maintaining remission along with reduced Mayo Clinic disease index and improved gut microbiota	[105]
	Symprove ™ (*L. rhamnosus* NCIMB 30174, *L. plantarum* NCIMB 30173, *L. acidophilus* NCIMB 30175 and *Enterococcus faecium* NCIMB 30176)	Liquid; 1 mL/kg each morning on a fasted stomach for 4 weeks	Significantly reduced levels of fecal calprotectin along with decreased intestinal inflammation in patients	[106]
	VSL#3, *L. reuteri* DSM 17938 and *S. thermophilus*, *L. acidophilus*, *B. breve* and *B. animalis* ssp. *lactis*	Variable/day for 12 months	Marked reduction in total adverse events along with decreased need for systemic steroids, hospitalization, and surgery	[107]
Crohn’s Disease	VSL # 3 (4 x e *Lactobacillus,* 3 x *Bifidobacterium*, and 1 x *S. thermophilus*)	Sachets; x 2/day for 90 days	No statistical difference between VSL#3 and placebo treatment	[108]
Colon cancer (CRC)	*L. acidophilus* LA-5, *L. plantarum*, *B. lactis* BB-12, *S. boulardii*	Capsule; x 2/day for 16 days	Decreased the risk of postoperative complications	[109]
	*L. plantarum* CGMMCC nr 1258, *L. acidophilus* LA-11, *B. longum* BL-88	Capsule; 1/day for 6 days preoperatively and 10 days post-operatively	Improvement in the integrity of gut mucosal barrier and decrease in infections complications	[110]
	*L. casei, L. acidophilus, L. lactis, B. bifidum, B. longum, B. infantis*	Sachets; x 2/day for 4 weeks	Improved the quality of life and inflammatory status of the CRC patients	[111]
	*B. lactis, L. acidophilus*	Tablets; x 2/day	Improved the diversity and abundance of butyrate-producing bacteria (*Clostridiales* and *Faecalibacterium* species) in fecal and mucosal microbiota of CRC patients Significant reduction of *Fusobacterium* and *Peptostreptococcus* species in fecal microbiota of CRC patients	[112]
*H. pylori* infection	*L. Reuteri*	Chewable tablets/day for 4 weeks	Effectively suppresses *H. pylori* infection and decreases the occurrence of dyspeptic symptoms Does not seem to affect antibiotic therapy outcome	[113]
	*Lactobacillus*	2 weeks	High rate of eradication	[114]

**Table 2:** Prebiotics in intestinal disorder.

Intestinal Disorder	Prebiotic/Synbiotic	Administration Method and Duration	Results	Reference
Constipation	Orafti^®^ GR Inulin (inulin from chicory) or Maltodextrin DE 19 (maltodextrin)	Sachets (4 g inulin or maltodextrin/sachet); x 3/day for 4 weeks	A significant increase of stool frequency was documented, which was accompanied by a softening of stool consistency, which had a positive impact on the quality of life, primarily increasing the satisfaction	[115]
Irritable bowel syndrome (IBS)	Short-chain fructo-oligosaccharide	Powder; 5 g/day for 4 weeks	Rectal discomfort threshold and IBS and quality of life scores were significantly improved	[116]
	Pectin powder	Powder; 26 g/day for 6 weeks	Pectin acts as a prebiotic in specifically stimulating gut bifidobacteria in IBS-diarrhea patients and is effective in alleviating clinical symptoms, balancing colonic microflora, and relieving systemic inflammation. In view of its ability to re-establish a healthy gut ecosystem, pectin has the potential of being a therapeutic agent in IBS-diarrhea	[117]
	*L. acidophilus* La-5^®^ and *Bifidobacterium* BB-12^®^ and Beneo dietary fibres	Fermented milk (180 g) x 2/day for 4 weeks	On average, an 18% improvement in total IBS-QoL score was reported and significant improvements in bloating severity, satisfaction with bowel movements, and the severity of IBS symptoms’ interference with patients’ everyday life were observed. However, there were no statistically significant differences between the synbiotic group and the placebo group	[118]
Ulcerative colitis (UC)	*Enterococcus faecium, L. plantarum, S. thermophilus, B. lactis, L.acidophilus, B. longum*, and fructooligosaccharide	Chewable tablets; x 2/day for 8 weeks	Overall, 55.6% of patients attained remission, while improved clinical activity index and reduction in C-reactive protein and sedimentation values were observed	[119]
	*Streptococcus faecalis* T-110 JPC, *Clostridium butyricum* TO-A, *Bacillus mesentricus* TO-A JPC, *L. sporogenes* plus prebiotic	Capsule; x 2/day for 3 months	Remission	[120]
Colon cancer	*Pediococcus pentosaceus, Leuconostoc mesenteroides, L. paracasei, L. plantarum* (10 × 10^11^ CFU) and inulin, resistant starch, pectin, and b-glucan (2.5 g)	Synbiotic sachets; (12 g)/day	Increased gastrointestinal Quality of Life index Improved the functional bowel disorder score	[121]
	*L acidophilus, L rhamnosus, L paracasei, B lactis*, and fructo-oligosaccharides	Sachets; x 2/day for 19 days	Decreases postoperative infections	[122]
	Prebiotic supplement: fructooligosaccharide (25%), xylooligosaccharide (25%), polydextrose (25%), and resistant dextrin (25%)	30 g/day for 7 days	Improved serum immunologic indicators in patients with CRC 7 d before operation Changed the abundance of four commensal microbiota (Bacteroides, Bifidobacterium, Escherichia-Shigella, and Enterococcus), and Escherichia-Shigella, Bacteroides, and Enterococcus are the genera known to include pathogenic strains	[123]
H. pylori	*B. lactis* B94 (5 × 10^9)^ CFU/dose), inulin (900 mg/dose)	Sachets; x 3/day for 14 days	From a total of 69 H. pylori-infected children, eradication was achieved in 20 out of 34 participants in the standard therapy group and 27/35 participants in the synbiotic group. There were no significant differences in eradication rates between the standard therapy and the synbiotic groups	[124]
